# Comparison of Postoperative Analgesic Efficacy of Two Intrathecal Morphine Doses (75 μg vs 100 μg) for Cesarean Delivery: A Double-Blind Randomized Study

**DOI:** 10.7759/cureus.107761

**Published:** 2026-04-26

**Authors:** Joseph A Veraza, Mariam A Martinez, Pedro Angulo, Mabel Z Almeida

**Affiliations:** 1 Anesthesiology, Clínica Andes Salud Puerto Montt, Puerto Montt, CHL; 2 Anesthesiology, Hospital Universitario de Caracas, Caracas, VEN; 3 Biostatistics, Hospital Universitario de Caracas, Caracas, VEN

**Keywords:** anesthesia spinal, elective cesarean delivery, intrathecal morphine, obstetric anesthesia, postoperative pain

## Abstract

Background: Intrathecal morphine remains a cornerstone of post-cesarean analgesia due to its prolonged spinal effect; however, its use is limited by dose-dependent adverse effects. Contemporary practice trends favor dose minimization, yet the lowest dose that reliably maintains effective analgesia remains unclear. This study aimed to evaluate whether a reduced dose of 75 μg provides analgesic efficacy comparable to a standard 100 μg dose.

Methods: In this prospective, randomized, double-blind trial, 240 ASA II women undergoing elective cesarean delivery under spinal anesthesia were allocated to receive either 75 μg or 100 μg of preservative-free intrathecal morphine combined with 7.5 mg of 0.5% isobaric bupivacaine. Postoperative pain intensity was assessed using the Visual Analog Scale (VAS, 0-10) at 0, 4, 8, 12, and 24 hours. Secondary outcomes included rescue analgesic requirements, maternal adverse effects, and neonatal Apgar scores.

Results: All 240 enrolled participants completed the study protocol. At the primary 12-hour endpoint, pain intensity did not differ significantly between groups, with median VAS scores of 0.0 (IQR 1.0) in the 75 μg group and 1.0 (IQR 1.75) in the 100 μg group (p = 0.224); no significant differences were observed at 4, 8, or 24 hours. Non-opioid rescue analgesia was required in 50/120 (41.7%) patients in the 75 μg group and 45/120 (37.5%) in the 100 μg group (p = 0.509), with no patients requiring opioid rescue. Maternal adverse effects were comparable between groups, including pruritus in 30/120 (25.0%) patients in the 75 μg group and 25/120 (20.8%) in the 100 μg group (p = 0.442), and nausea in 20/120 (16.7%) and 10/120 (8.3%) patients, respectively (p = 0.051). Neonatal outcomes were favorable, with all neonates achieving Apgar scores ≥8 at five minutes. Although statistically significant differences were observed between groups, these differences were not clinically meaningful.

Conclusions: A 75 μg dose of intrathecal morphine was associated with comparable 24-hour postoperative analgesia to 100 μg, with no statistically significant differences observed between groups in pain scores or adverse effects. These findings suggest that lower doses may provide adequate analgesia while maintaining a similar safety profile. However, further studies specifically designed as non-inferiority or equivalence trials are required to confirm these observations.

## Introduction

Effective pain control after cesarean delivery plays a central role in early mobilization, maternal comfort, and successful initiation of breastfeeding [1]. Among neuraxial adjuncts, intrathecal morphine remains the cornerstone of post-cesarean analgesia due to its hydrophilic properties and prolonged spinal effect [2]. However, its clinical utility is tempered by dose-dependent adverse effects, most commonly pruritus, nausea, and vomiting, and less frequently respiratory depression [3-5].

Traditionally, intrathecal morphine doses of 100 μg or higher were routinely administered. Over time, accumulating evidence has suggested that lower doses may provide adequate analgesia while reducing opioid-related side effects [4]. While ultra-low doses (e.g., 50 μg) significantly minimize side effects, they often fail to provide sustained 24-hour analgesic coverage. Therefore, we hypothesized that an intermediate dose of 75 μg represents an optimal clinical balance, bridging the quantified research gap between analgesic efficacy and side effect profile.

The present study was designed to compare the analgesic efficacy and safety of two intrathecal morphine doses, 75 μg and 100 μg, in women undergoing elective cesarean delivery under spinal anesthesia. We hypothesized that the lower dose would provide comparable analgesia during the first 24 postoperative hours without compromising maternal or neonatal outcomes.

## Materials and methods

Study design and ethical compliance

This prospective, randomized, double-blind trial was conducted at the University Hospital of Caracas. The study protocol was approved by the Institutional Review Board (IRB No. HUC-ANEST-2024-2045), and written informed consent was obtained from all participants. In accordance with local regulations at the time of patient enrollment, the study adhered strictly to the ethical principles of the Declaration of Helsinki. The study was registered at ClinicalTrials.gov (Identifier: NCT07516717). Registration was completed retrospectively due to administrative timing considerations.

Participants

Women with ASA physical status II scheduled for elective cesarean delivery under spinal anesthesia were eligible for inclusion. Exclusion criteria included contraindications to neuraxial anesthesia, known hypersensitivity to opioids or local anesthetics, multiple gestation, hypertensive disorders of pregnancy, body mass index greater than 30 kg/m², and the presence of significant systemic disease.

Sample size justification

The sample size was calculated based on the primary outcome of postoperative pain intensity. Assuming a clinically significant difference of 1.0 point on the Visual Analog Scale (VAS) between groups, with a standard deviation of 1.5, an alpha error of 0.05, and a power of 80%, approximately 100 patients per group were required. To account for potential dropouts, 120 patients per group were enrolled, totaling 240 participants.

Randomization and blinding

Participants were randomly allocated in a 1:1 ratio using a computer-generated randomization sequence. Group assignments were concealed using the Sequentially Numbered, Opaque, Sealed Envelopes (SNOSE) method. An anesthesiologist not involved in intraoperative management prepared the intrathecal solutions, ensuring blinding of patients, anesthesia providers, and outcome assessors. A successful blinding assessment was confirmed postoperatively.

Anesthetic management

Spinal anesthesia was performed at the L3-L4 interspace using a 25 G or 27 G pencil-point needle. All patients received 7.5 mg of 0.5% isobaric bupivacaine combined with either 75 μg (Group A) or 100 μg (Group B) of intrathecal morphine. The rationale for utilizing 7.5 mg of isobaric bupivacaine was to guarantee an adequate T4 sensory level while minimizing maternal hypotension. No intraoperative systemic opioid supplementation was administered.

Outcome measures

The primary outcome was postoperative pain intensity measured using the Visual Analog Scale (VAS, 0-10) [6] at 12 hours, with assessments at 0, 4, 8, and 24 hours. Secondary outcomes included rescue analgesia requirements, maternal adverse effects, and neonatal well-being assessed using Apgar scores [7] at one and five minutes. Sedation levels were monitored using the Ramsay Sedation Scale [8]. Clinically significant respiratory depression was predefined as a respiratory rate < 10 breaths/min.

Statistical analysis

Data analysis was performed using PAST statistical software version 2.17c (Øyvind Hammer, Natural History Museum, University of Oslo, Oslo, Norway). Normality was assessed using the Kolmogorov-Smirnov test. Continuous variables are presented as mean ± SD or median (IQR), and categorical variables as frequencies and percentages. Between-group comparisons were performed using Student’s t-test or Mann-Whitney U test for continuous variables, and chi-square or Fisher’s exact test for categorical variables. A p-value < 0.05 was considered statistically significant. Corresponding test statistics are reported in the tables.

## Results

A total of 240 participants were assessed for eligibility and randomized, with all subjects completing the study protocol. Baseline demographic characteristics, including age, ASA physical status, and operative duration, were comparable between the two groups (Table 1).

**Table 1:** Baseline demographic characteristics *Calculated using independent Student’s t-test. **Calculated using chi-square test.

Characteristic	Group 75 μg (n = 120)	Group 100 μg (n = 120)	Test statistic	p-value
Age (years), mean ± SD	29.17 ± 7.28	28.88 ± 7.15	t = 0.311	0.765*
ASA physical status II, n (%)	120 (100.0%)	120 (100.0%)	χ² = 0.00	>0.99**

Primary and secondary pain outcomes

Postoperative pain scores showed an early difference between groups (Table 2). Immediately postoperatively (0 hours), the 100 μg group demonstrated lower pain scores compared to the 75 μg group (p < 0.001). However, at the primary 12-hour endpoint, no statistically significant difference was observed between groups, with median VAS scores of 0.0 (IQR 1.0) in the 75 μg group and 1.0 (IQR 1.75) in the 100 μg group (p = 0.224). Similarly, pain scores at 4, 8, and 24 hours did not differ significantly between groups (Table 2).

**Table 2:** Postoperative pain scores (Visual Analog Scale) *p-values were calculated using the Mann-Whitney U test (standardized Z-score). Data are presented as median (IQR).

Time of measurement	Group 75 μg (n = 120)	Group 100 μg (n = 120)	Test statistic	p-value*
0 hours	1.5 (2.0)	1.0 (3.0)	Z = 4.12	<0.001
4 hours	3.0 (3.75)	4.0 (4.0)	Z = 1.93	0.053
8 hours	2.0 (3.0)	1.0 (3.0)	Z = 0.72	0.472
12 hours	0.0 (1.0)	1.0 (1.75)	Z = 1.21	0.224
24 hours	0.0 (0.0)	0.0 (1.0)	Z = 0.00	>0.999

Analgesic consumption and adverse effects

Non-opioid rescue analgesia (nonsteroidal anti-inflammatory drugs, NSAIDs) was required in 50 (41.7%) patients in the 75 μg group and 45 (37.5%) in the 100 μg group (p = 0.509). No patients required opioid rescue analgesia. Maternal adverse effects occurred at similar frequencies between groups (Table 3), including pruritus (25.0% vs 20.8%, p = 0.442), nausea (16.7% vs 8.3%, p = 0.051), and vomiting (4.2% vs 8.3%, p = 0.182).

**Table 3:** Rescue analgesia and maternal adverse effects *p-values were calculated using the chi-square test. Rescue analgesia consisted of intravenous ketorolac. No additional rescue medications were administered. NSAIDs: nonsteroidal anti-inflammatory drugs.

Variable	Group 75 μg (n = 120)	Group 100 μg (n = 120)	Test statistic	p-value*
Rescue analgesia				
Non-opioid (NSAIDs) ketorolac, n (%)	50 (41.7%)	45 (37.5%)	χ² = 0.436	0.509
Adverse effects				
Pruritus, n (%)	30 (25.0%)	25 (20.8%)	χ² = 0.590	0.442
Nausea, n (%)	20 (16.7%)	10 (8.3%)	χ² = 3.810	0.051
Vomiting, n (%)	5 (4.2%)	10 (8.3%)	χ² = 1.770	0.182

Neonatal outcomes

Neonatal outcomes were favorable in both groups (Table 4), with all neonates achieving Apgar scores ≥8 at five minutes. Although statistically significant differences in Apgar scores were observed between groups (p < 0.001), these differences were not clinically meaningful.

**Table 4:** Neonatal outcomes (Apgar scores) *p-values were calculated using the Mann-Whitney U test (standardized Z-score). Data are presented as median (IQR).

Assessment time	Group 75 μg (n = 120)	Group 100 μg (n = 120)	Test statistic	p-value*
APGAR at 1 minute	8.0 (1.0)	8.0 (0.0)	Z = 3.85	<0.001
APGAR at 5 minutes	9.5 (1.0)	10.0 (0.75)	Z = 4.22	<0.001

No patient in either group experienced clinically significant respiratory depression or sedation requiring intervention.

## Discussion

This study suggests that a reduced intrathecal morphine dose of 75 μg may provide adequate postoperative analgesia over a 24-hour period, with no statistically significant differences observed compared to the 100 μg dose at most assessed time points. Although the 100 μg dose was associated with lower pain scores immediately postoperatively (0 hours), this difference was transient and did not persist at later assessments. These findings are consistent with the Enhanced Recovery After Surgery (ERAS) guidelines, which emphasize opioid-sparing multimodal analgesia to facilitate maternal recovery [9-11]. In contrast to studies favoring higher intrathecal morphine doses to ensure early postoperative analgesia, our results suggest that reduced dosing may still achieve effective pain control when incorporated into a multimodal postoperative regimen.

Our findings regarding the 75 μg dose align with recent investigations exploring the lowest effective dose of intrathecal morphine [10]. For instance, previous studies have reported that while 100 μg provides reliable analgesia, intermediate doses between 50 μg and 75 μg may reduce the incidence of opioid-related adverse effects, such as pruritus and nausea, without substantially altering postoperative pain scores at 24 hours [12]. Furthermore, the use of intermediate intrathecal morphine doses in conjunction with multimodal systemic analgesia, such as the routine administration of NSAIDs in our cohort, has been shown to attenuate early postoperative differences in pain intensity often observed when comparing lower doses to the traditional 100 μg standard [2].

Importantly, although statistically significant differences in Apgar scores were observed between groups, all neonates achieved clinically reassuring values (≥8 at five minutes), indicating that these differences were not clinically meaningful.

Limitations

This study has several limitations. First, the study population was restricted to ASA II obstetric patients, which may limit the generalizability of the findings to higher-risk populations. Second, the use of 7.5 mg of intrathecal bupivacaine reflects an institutional protocol tailored to the anthropometric characteristics of our patient population. In spinal anesthesia, local anesthetic requirements are influenced by factors such as patient height and intrathecal spread; therefore, lower doses may be sufficient in populations with lower average height. However, this may limit the direct generalizability of our findings to populations in which higher doses are routinely used. Third, the study was not designed as a non-inferiority or equivalence trial; therefore, the absence of statistically significant differences should not be interpreted as evidence of equivalence. Finally, long-term outcomes beyond 24 hours were not assessed, limiting conclusions regarding prolonged analgesic efficacy and delayed adverse effects.

## Conclusions

In women undergoing elective cesarean delivery under spinal anesthesia, a 75 μg dose of intrathecal morphine was associated with postoperative analgesia without statistically significant differences compared to the 100 μg dose at most time points, and with no significant differences in maternal or neonatal safety outcomes. These findings suggest that lower intrathecal morphine doses may be adequate within multimodal analgesic protocols. However, as this study was not designed as a non-inferiority or equivalence trial, definitive conclusions regarding equivalence cannot be established.
